# Mapping Gastroesophageal Reflux Disease and Coronary Artery Disease: A Comprehensive Analysis of Multivariable Mendelian Randomization and Shared Genetic Etiology

**DOI:** 10.1002/clc.70213

**Published:** 2025-10-21

**Authors:** Yiying Zhen, Xiang Yuan, Min Ruan, Huan Lu, Dakai Liang, Dehua Huang, Fengyang Deng, Haozhang Huang, Jiaman Ou

**Affiliations:** ^1^ Department of Cardiology People′s Hospital of Yangjiang Yangjiang China; ^2^ Department of Cardiology Guangdong Cardiovascular Institute, Guangdong Provincial People′s Hospital (Guangdong Academy of Medical Sciences) Southern Medical University Guangzhou China; ^3^ Guangdong Provincial Key Laboratory of Coronary Heart Disease Prevention Guangdong Provincial People′s Hospital, Guangdong Academy of Medical Sciences Guangzhou China

**Keywords:** colocalization, coronary artery disease, gastroesophageal reflux disease, genetic correlation, multivariable Mendelian randomization

## Abstract

**Aims:**

We employed a robust genetic approach to provide a better understanding of whether Gastroesophageal reflux disease (GERD) contributes to coronary artery disease (CAD) risk from a genetic perspective.

**Methods:**

Multivariable Mendelian Randomization (MVMR) was applied to explore causal links between GERD and CAD using genetic instruments derived from genome‐wide association studies (GWAS). The MVMR models were adjusted for key metabolic confounders, including low‐density lipoprotein cholesterol (LDL‐C), body mass index (BMI), systolic blood pressure (SBP), and glycated hemoglobin (HbA1c). Genetic correlations were estimated using linkage disequilibrium score regression. Cross‐trait meta‐analyses, Heritability Estimation from Summary Statistics (ρ‐HESS) and colocalization analyses were performed to identify pleiotropic genes and shared genetic loci, elucidating the genetic relationship between GERD and CAD.

**Results:**

Genetically predicted GERD was found to be causally linked with CAD (rg = 0.38, *P* = 2.37E‐52), independent of metabolic risk factors, including LDL‐C, BMI, SBP, and HbA1c (odds ratio: 1.24, 95% CI: 1.02–1.52, *p* < 0.05). Cross‐trait meta‐analyses identified eight novel pleiotropic single nucleotide polymorphisms, four of which were independent of metabolic confounders, including rs11764337 in MAD1L1, rs2240326 in RBM5, rs9615905 in FAM19A5, and rs9837341 in BSN. ρ‐HESS and colocalization analysis further revealed shared genetic loci for GERD and CAD, specifically rs4643373 in IGF2BP1 (located in chr17: 45876022‐47517400 and posterior probability for H4 > 0.75).

**Conclusions:**

GERD is identified as an independent risk factor for CAD. The discovery of shared genetic loci provides novel insights into the genetic mechanisms underlying GERD and CAD, with IGF2BP1 emerging as a potential therapeutic target for intervention.

## Summary

**What is known?**

- GERD and CAD share common risk factors, but their genetic relationship is unclear.

**What is new?**

- This study provides novel evidence of a causal relationship between GERD and CAD using multivariable Mendelian randomization. It identifies shared genetic loci, such as IGF2BP1 and FAM19A5, that may contribute to both conditions.

**What is next?**

- Future research should explore the biological pathways linking GERD and CAD, focusing on inflammation, and assess if managing GERD reduces cardiovascular risk.

## Graphical abstract

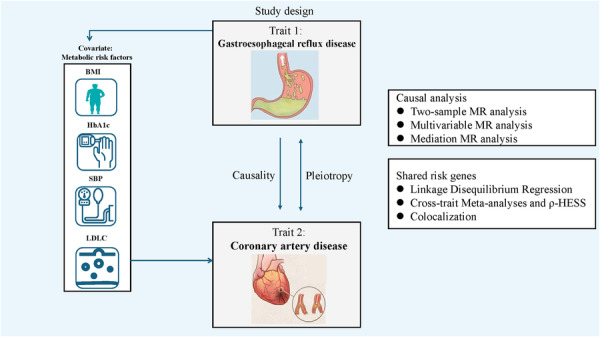

**Abbreviations**
BMI: body mass index
CAD: coronary artery disease
GERD: gastroesophageal reflux disease
GWAS: genome‐wide association studies
HbA1c: glycated hemoglobin
LDL‐C: low‐density lipoprotein cholesterol
MVMR: multivariable Mendelian randomization
SBP: systolic blood pressure
SNPs: single nucleotide polymorphisms

## 1 Introduction

Gastroesophageal reflux disease (GERD) is a chronic digestive disorder that occurs when stomach acid or, occasionally, bile flows back (refluxes) into the esophagus. Estimates suggest that approximately 20%–30% of the adult population may experience GERD symptoms [1]. Interestingly, among individuals with GERD, the prevalence of coronary artery disease (CAD) is notably higher than in the general population [2]. Studies in elderly populations often report coexistence rates higher than those in younger groups [3]. However, as of the current state of medical research, there is no definitive evidence to declare a direct causal relationship between GERD and CAD. Therefore, more robust, well‐designed studies are needed to explore whether there is any direct causal link between GERD and CAD.

The current medical understanding is that GERD and CAD share several common risk factors, such as obesity, smoking, poor diet, and a sedentary lifestyle [3]. These factors can independently contribute to the development of each condition, which might explain their co‐occurrence in many patients. Besides, chronic inflammation and oxidative stress may be the underlying mechanisms that link the GERD and CAD [4]. However, these are common pathways in many diseases and do not establish direct causality between GERD and CAD. Most studies exploring the relationship between GERD and CAD are observational [5], which can identify associations but not establish causality. Longitudinal studies that follow patients over time are needed to better understand the causal relationship, but these studies are expensive and time‐consuming. Given the prevalence and substantial health burden of both GERD and CAD, elucidating causal relationships could have significant implications for prevention and treatment strategies.

Multivariable Mendelian Randomization (MVMR) offers a powerful tool to explore the association between GERD and CAD, effectively addressing key limitations inherent in traditional observational studies. MVMR uses genetic variants (single nucleotide polymorphisms, or SNPs) as instrumental variables to infer causality. By reducing confounding bias, addressing reverse causation, and improving causal inference, MVMR enables the derivation of more definitive conclusions regarding the potential causal relationship between these conditions [6]. Furthermore, this approach can unravel shared biological pathways, providing critical insights that may inform the identification of novel therapeutic targets. These insights hold the potential to significantly advance the prevention and treatment strategies for patients affected by GERD, CAD, or both, thereby improving clinical outcomes.

This study aims to elucidate the causal relationship between GERD and CAD. To achieve this, we employed MVMR to infer causality, a method that effectively mitigates confounding and reverse causation. Leveraging data from large‐scale GWAS, we assessed the potential causal effect of GERD on CAD. Furthermore, we investigated shared genetic targets between these diseases to uncover the underlying pathways and mechanisms that may drive their association, providing new insights into the genetic mechanisms linking these conditions and identifying potential targets for therapeutic intervention.

## 2 Methods

### 2.1 Study Design

This study employed a MVMR approach to investigate the causal relationship and shard risk genes between GERD and CAD (Central Illustration 1, 1). All data handling and analyses were conducted in accordance with STROBE‐MR guidelines [7].

**Central Illustration 1:**
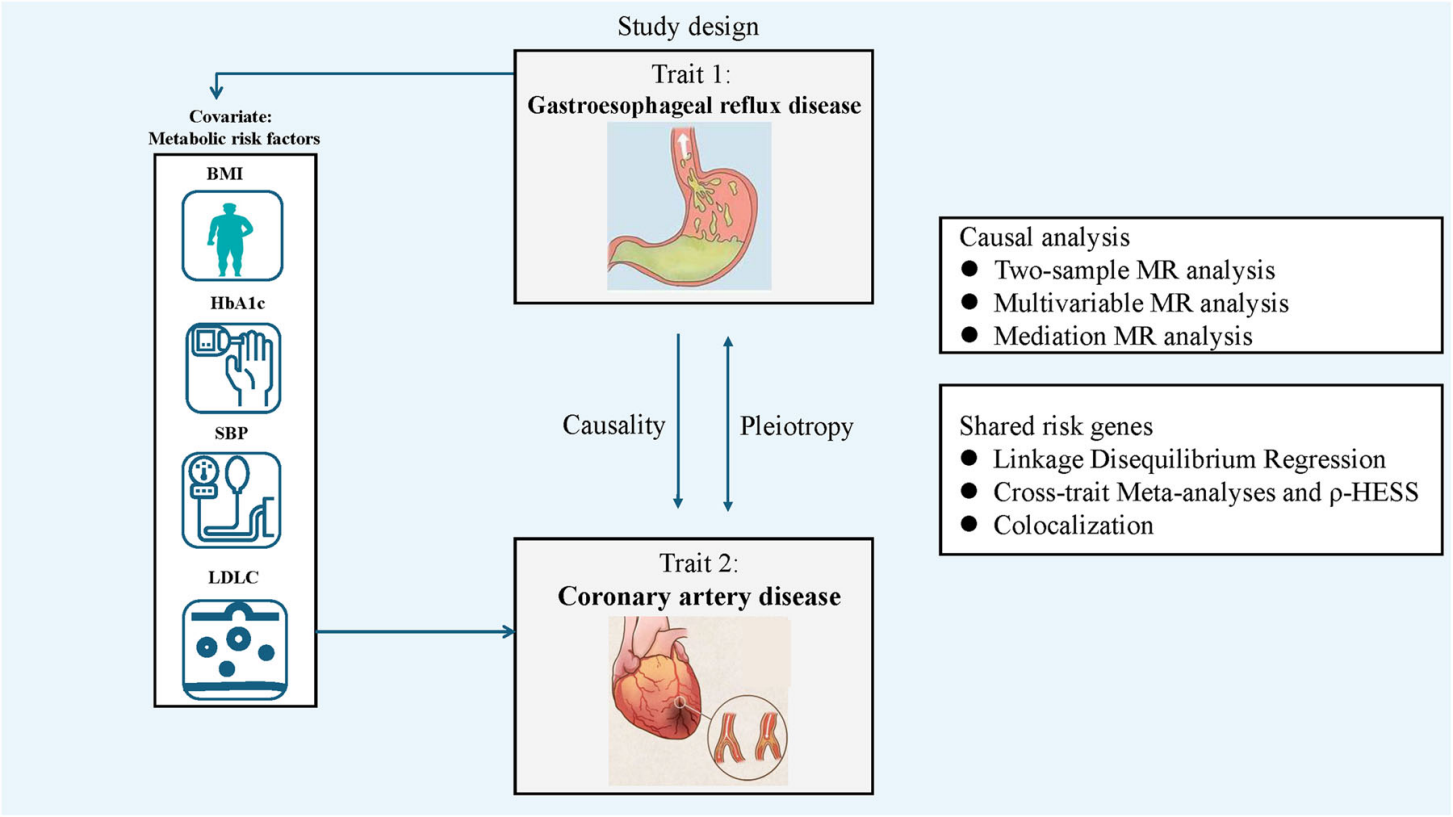
Central Illustration 1. Study Design.

### 2.2 Data Sources of Exposure and Outcome

The GERD exposure data set was derived from the study by Ong et al. which utilized a multi‐trait genome‐wide association study (GWAS) model [8]. This model incorporated data on BMI, major depressive disorder, educational attainment, GERD, and Barrett′s esophagus to uncover additional susceptibility loci associated with GERD. The data set, consisting of 129 080 cases and 473 524 controls of European ancestry, was obtained directly from the GWAS Catalog (https://www.ebi.ac.uk/gwas/studies/GCST90000514) and is summarized in Table 1.

**Table 1:** Overview of the GWAS data used in the study.

Phenotype	Sample size (case/control)	Ancestry	Consortium or cohort study	Year of publication or release	PubMed identifier
**Outcome (*n*** = **4)**					
Coronary atherosclerosis	23 363/187 840	European	FinnGen	2023	36653562
CAD	60 801/123 504	77% European	CARDIoGRAMplusC4D	2015	26343387
MI	43 676/128 199	77% European	CARDIoGRAMplusC4D		
**Exposure**					
GERD	129 080/473 524	European	UK Biobank, 23andMe	2021	34187846
**Mediator (*n*** = **4)**					
BMI	681 275	European	GIANT	2018	30124842
LDL‐C	440 546	European	UK Biobank	2020	32203549
HbA1c	88 355	European	MAGIC	2017	28898252
SBP	757 601	European	ICBP, UK Biobank	2018	30224653

Abbreviation: AMI, Acute myocardial infarction; BMI, Body mass index; CAD, Coronary artery disease; CERD, Gastroesophageal reflux disease; LDLC, Low‐density lipoprotein cholesterol.

Outcome data for coronary artery atherosclerotic disease were sourced from the FinnGen project, a large‐scale initiative integrating genetic and health registry information from approximately 500 000 participants of Finnish descent [9]. The FinnGen data set provided meta‐analytic GWAS results for 3095 phenotypes, including 16 243 cases of atherosclerosis and 381 977 controls, as well as 51 589 cases of coronary atherosclerosis and 343 079 controls, all of European ancestry. Additional data on CAD (60 801 cases and 123 504 controls) and MI (43 676 cases and 128 199 controls) were retrieved from the CARDIoGRAMplusC4D Consortium [10].

### 2.3 Genetic Instruments

Genetic variants associated with GERD were identified from GWAS datasets. Instrumental variables were selected based on a strong association with GERD (*P* < 5E‐08) and were pruned to ensure independence by avoiding linkage disequilibrium (LD) (R^2^ < 0.001 within a 10 000 KB window). Only SNPs with a minor allele frequency (MAF) greater than 0.01 were retained, and those with insufficient F‐statistics were excluded to maintain the validity of the instrumental variables. To mitigate potential reverse causation, Steiger filtering was applied. Genetic instruments for coronary artery atherosclerotic disease and its subtypes were similarly selected from the respective GWAS datasets.

### 2.4 Statistical Analysis

#### 2.4.1 Heritability and Genetic Correlation

To estimate the genetic correlation between GERD and coronary atherosclerotic diseases, linkage disequilibrium score regression (LDSC) was performed [11]. This analysis utilized precomputed linkage disequilibrium (LD) data from the 1000 Genomes Project, specifically focusing on SNPs included in the HapMap 3 SNP set. To enhance the reliability of the results, SNPs that were misaligned with the reference panel were excluded from the analysis [12]. GWAS summary statistics, along with LD scores derived from European ancestry reference data in the 1000 Genomes Project, were used to conduct the LDSC analysis. The threshold for statistical significance in the genetic correlation assessment was defined as *p* < 0.05.

#### 2.4.2 Multivariable Mendelian Randomization

To investigate the relationship between GERD, metabolic risk factors, and coronary atherosclerotic disease, we first applied bi‐direction two‐sample MR and mediation MR analyses. These preliminary steps explored the potential associations between GERD and metabolic risk factors, as well as their impact on coronary atherosclerosis. Subsequently, MVMR analysis was conducted to estimate the independent causal effect of GERD on coronary atherosclerotic disease. This approach allows for the inclusion of multiple exposures, enabling the adjustment for confounding factors such as LDL‐C, BMI, SBP, and HbA1c. The following steps were performed: Independent SNPs strongly associated with GERD and relevant confounding factors were identified and selected as genetic instruments. These instruments were then harmonized to ensure consistency in the direction of their effects on GERD, the confounding factors, and coronary atherosclerosis phenotype outcomes across all datasets. A MVMR analysis was conducted, with causal estimates derived using the inverse‐variance weighted (IVW) method. To ensure the robustness of the results, additional sensitivity analyses were performed, including MR‐lasso, MR‐Egger, and weighted median approaches.

#### 2.4.3 Cross‐Trait GWAS Meta‐Analysis and Colocalization Analysis

To identify shared genetic risk factors between GERD and CAD, two complementary approaches were utilized: Multi‐Trait Analysis of GWAS (MTAG) and Cross‐Phenotype Association (CPASSOC) [13, 14, 15]. MTAG improves the estimation of SNP effects by incorporating multiple correlated traits into the analysis, enhancing the likelihood of detecting loci associated with any of the included traits. This method assumes that SNPs exhibit varying degrees of heritability across traits and that there is a positive, albeit imperfect, genetic covariance among them. To test the assumption of an equal variance‐covariance structure for shared SNP effect sizes across traits, a maximum allowable false discovery rate (‘maxFDR’) was applied [13]. MTAG improves the estimation of SNP effects by incorporating multiple correlated traits into the analysis, enhancing the likelihood of detecting loci associated with any of the included traits. This method assumes that SNPs exhibit varying degrees of heritability across traits and that there is a positive, albeit imperfect, genetic covariance among them. To test the assumption of an equal variance‐covariance structure for shared SNP effect sizes across traits, a maximum allowable false discovery rate (‘maxFDR′) was applied [16].

Significant SNPs were identified as those reaching genome‐wide significance (*P* < 5E‐08) in both MTAG and CPASSOC analyses. SNP clumping was performed using PLINK with the following parameters: ‐clump‐p1 5E‐08, ‐clump‐p2 1E‐05, ‐clump‐r² 0.2, and ‐clump‐kb 500. Novel loci were defined as independent SNPs without linkage disequilibrium (LD; r² > 0.2 within a 1000‐kb window) with previously identified loci from single‐trait GWAS results.

Colocalization analysis was conducted using the *coloc* method, with loci considered colocalized if the posterior probability for hypothesis 4 (PPH4) exceeded 0.75. SNPs and their proxies in relevant populations were analyzed using LDlink to validate LD structures and confirm findings [17].

#### 2.4.4 Local Genetic Correlation Analysis

Heritability from summary statistics (ρ‐HESS) was used to evaluate local SNP heritability and genetic correlation [18]. We identified around 1703 approximately LD‐independent regions, each roughly 1.5 MB long. Local heritability and genetic interrelations were calculated based on the 1000 Genomes Project, with significance set at pval adjusted using Bonferroni correction < 0.05 (Pcorrection < 0.05).

## 3 Results

### 3.1 Bi‐Direction Causal Effects of CAD on GERD

**Figure 1:**
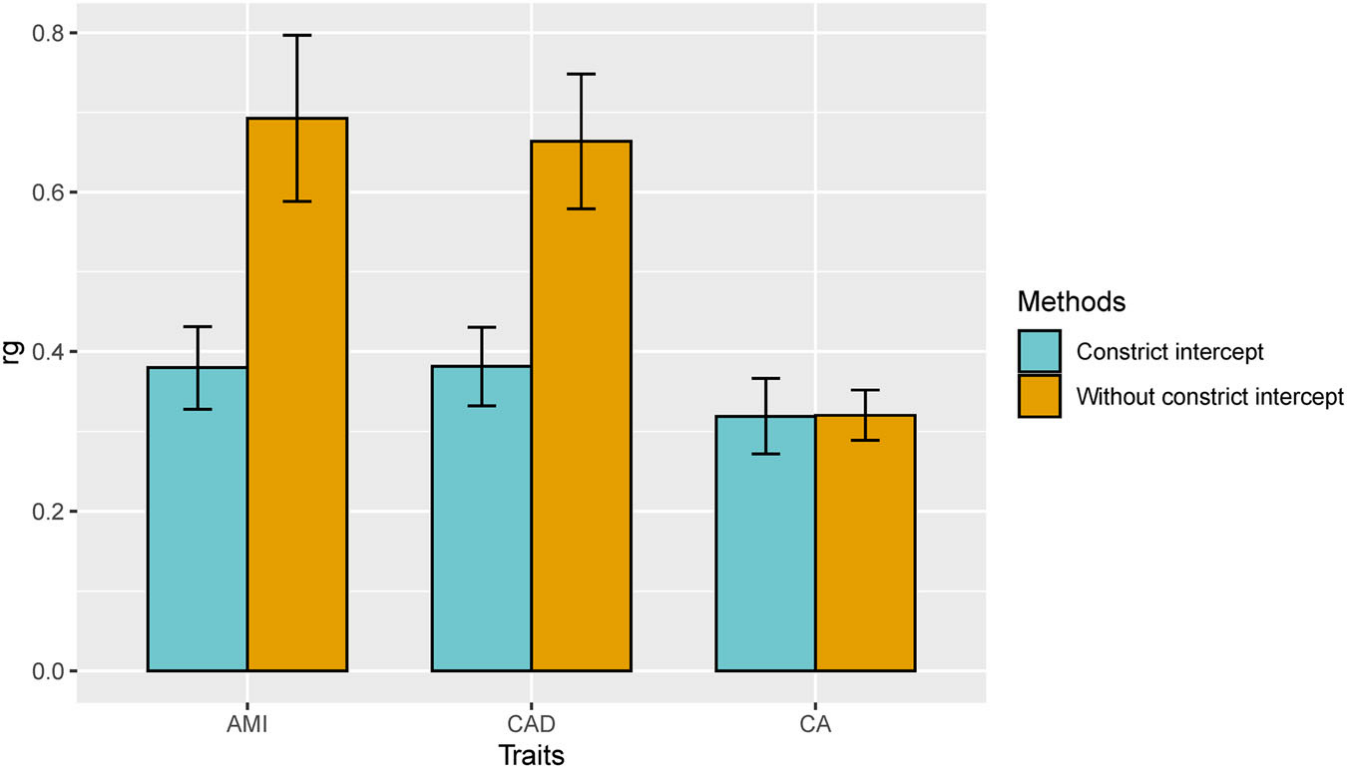
linkage disequilibrium score regression of GERD and coronary atherosclerosis phenotype. Abbreviation: AMI, Acute myocardial infarction; CAD, coronary artery disease; CA, coronary atherosclerosis.

**Figure 2:**
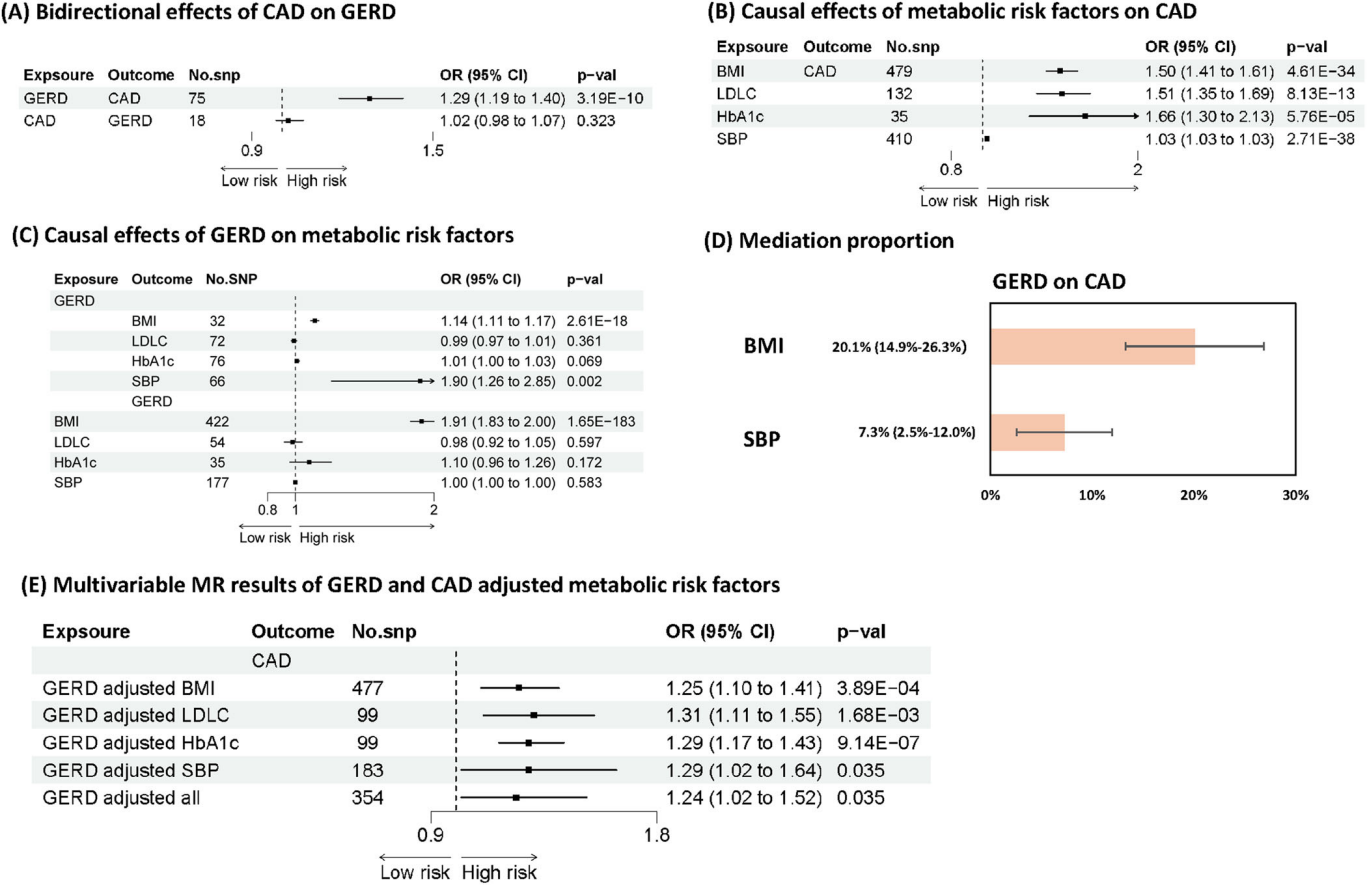
MR analysis of GERD, metabolic risk factors and CAD. The results were based on IVW methods. Abbreviation: BMI, Body mass index; CAD, Coronary artery disease; CERD, Gastroesophageal reflux disease; CI, Confidence interval; LDLC, Low‐density lipoprotein cholesterol; OR, Odds ratio; SBP, Systolic blood pressure. (A) Bidirectional effects of CAD on GERD. (B) Causal effects of metabolic risk factors on CAD. (C) Causal effects of GERD on metabolic risk factors. (D) Mediation proportion. (E) Multivariable MR results of GERD and CAD adjusted metabolic risk factors.

GERD was positively correlated with coronary atherosclerosis (rg = 0.32, *P* = 1.71E‐40), CAD (rg = 0.38, *P* = 2.37E‐52) and MI (rg = 0.38, *P* = 3.15E‐47), as determined by genetic correlation analysis (Figure 1, 1). Specific IVs are shown in Tables S1 and S2. Our analysis showed that GERD was significantly associated with increased risks of CAD (OR = 1.29, *P* = 3.19E‐10). The MR‐Egger intercept showed no evidence of directional pleiotropy (*p* > 0.05), and Cochran′s *Q* test indicated no evidence of heterogeneity (*p* > 0.05) (Figure 2A, Table S3 and Figure S1). No significant reverse causal effects were observed from CAD to GERD.

### 3.2 Impact of Metabolic Risk Factors

In our analysis, BMI, LDL‐C, SBP, and HbA1c were found to have a causal effect on CAD (Figure 2B). Among these, BMI and SBP were significant mediators in the relationship between GERD and CAD (Figure 2C,D). Specifically, BMI mediated 20.57% of the effect of GERD on CAD, and SBP mediated 7.25% of the GERD‐CAD association (Table S4). After adjusting for metabolic risk factors including BMI, LDL‐C, HbA1c, and SBP, GERD has independent causal effect on CAD (Figure 2E and Table S5).

### 3.3 Genetic Correlation and Shared Functional Genes

**Figure 3:**
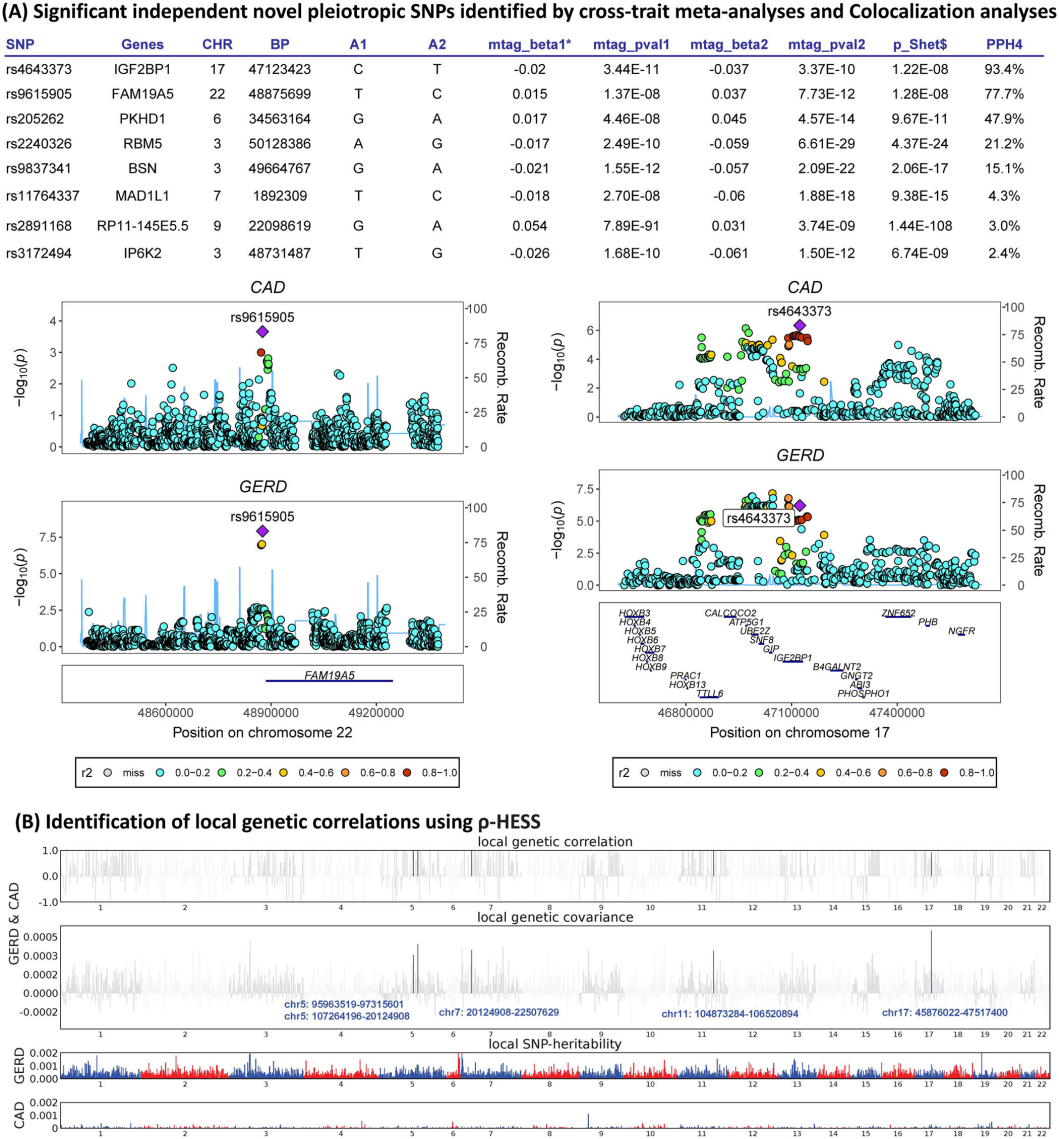
Shared genetic architecture between GERD and CAD. (A) *Based on Multi‐Trait Analysis of GWAS; $Based on Cross‐Phenotype Association; Beta1: beta of trait 1; Beta 2: beta of trait 2; Abbreviation: AMI, Acute myocardial infarction; CAD, Coronary artery disease; CERD, Gastroesophageal reflux disease; PPH4, probability for H4. (B) Manhattan plot showed the estimates of local genetic correlation and local genetic covariance between GERD and CAD, and local SNP heritability of GERD and CAD, respectively. Red and blue bars in “local genetic correlation” and “local genetic covariance” represent significant regions which shared SNP heritability, after multiple adjustment (P < 5E‐08 in both local SNP heritability test, and *p* < 0.05/1703 in local genetic covariance test).

After excluding SNPs significant in the single‐trait GWAS of GERD, CAD and MI, those in LD (r² ≥ 0.1), we identified eight novel pleiotropic SNPs (rs11764337, rs205262, rs2240326, rs2891168, rs3172494, rs4643373, rs9615905 and rs9837341) associated with the combined GERD‐CAD phenotype (Figure 3A). The SNPs rs11764337 were mapped to MAD1L1, rs205262 to PKHD1, rs2240326 to RBM5, rs2891168 to RP11‐145E5.5, rs3172494 to IP6K2, rs4643373 to IGF2BP1, rs9615905 to FAM19A5, and rs9837341 to BSN (Table S6). LD link analysis revealed significant associations of rs205262 with BMI [19], rs3172494 with BMI‐adjusted waist circumference and rs2891168 with type 2 diabetes mellit [20]. The remaining five SNPs (rs11764337, rs2240326, rs4643373, rs9615905, and rs9837341) likely represent novel pleiotropic loci for GERD and CAD, independent of metabolic risk factors (Table S7).

Additionally, cross‐trait meta‐analysis identified rs4643373 in IGF2BP1 and rs9615905 in FAM19A5 as shared loci for GERD and CAD with strong colocalization evidence (PPH4 > 0.75), indicating a shared causal variant (Figure 3A and Figures S2 and S3). After multiple corrections,a pronounced local correlation was identified in five regions, leading with chr17: 45 876 022–47 517 400, between GERD and CAD (*p* = 9.57E‐08, p‐adjust = 1.54E‐04) (Figure 3B and Table S8). Furthermore, ρ‐HESS and cross‐trait meta‐analysis identified one novel locus (rs3172494, located in IGF2BP1) shared between patients with GERD and CAD, and it showed evidence of colocalization (PPH4 > 0.95).

## 4 Discussion

Our analysis confirmed that genetically predicted GERD increases the risk of CAD, coronary atherosclerosis, and MI, even after adjusting for traditional cardiovascular risk factors like LDL‐C, BMI, SBP, and HbA1c. The MVMR analysis revealed a significant independent causal effect of GERD on CAD. BMI and SBP were found to mediate this relationship, while HbA1c and LDL‐C did not, highlighting the importance of targeting BMI and SBP to reduce CAD risk in GERD patients. However, no reverse causality from CAD to GERD was observed. Our cross‐trait meta‐analysis identified shared genetic loci, including rs4643373 in IGF2BP1 and rs9615905 in FAM19A5, which may serve as potential therapeutic targets for both GERD and CAD.

Some genetic studies have identified variants in the PKHD1 gene, such as rs205262, that may be associated with cardiovascular risk factors or conditions like CAD [21]. The exact mechanisms by which PKHD1 could influence CAD are not fully understood. However, it′s hypothesized that the gene′s role in cellular processes like proliferation, adhesion, and maintenance of ductal structures in the kidneys might also impact vascular health [22]. Our study uncovered a novel mechanism involving PKHD1 in the development of GERD, which has not been previously reported. The clinical significance of identifying the rs205262 variant in PKHD1 as a contributor may offer new targets for drug development, potentially leading to more effective treatments for GERD‐related CAD.

The rs9615905 is located within the FAM19A5 gene. FAM19A5 is a novel pro‐ and anti‐inflammatory adipocytokine that is encoded by the FAM19A5 gene. This gene is part of the TAFA family, and its protein has been shown to play a significant role in various vascular aging‐related pathologies, including atherosclerosis, cardio‐cerebral vascular diseases, and cognitive deficits [23, 24, 25]. This suggests that inflammation may be a key mechanism.

rs4643373, mapped to the IGF2BP1 gene, encodes a member of the insulin‐like growth factor 2 mRNA‐binding protein family. Its association with the risk of esophagogastric junction adenocarcinoma, gastric cancer, and related conditions has been extensively studied [26, 27, 28]. Although IGF2BP2 is classified as an oncofetal protein, its role in heart structure and function has also been widely explored, particularly in patients with CAD [29, 30, 31]. The IGF2BP family is upregulated in cardiomyocytes during cardiac stress and remodeling and returns to baseline levels in recovering hearts, reflecting its involvement in dynamic cardiac regulatory processes. Zhong et al. reported that GAS5 (Growth Arrest‐Specific 5) promotes glucose metabolic reprogramming and suppresses ferroptosis in endothelial progenitor cells via the IGF2BP1/SIX1 and miR‐23a‐3p/SLC7A11 dual regulatory pathways in CAD [30]. Inflammation may also play a critical role in this process, as previous studies have shown that IGF2BP1 is associated with NF‐κB activation and the production of pro‐inflammatory cytokines [32, 33]. The rs4643373 variant also shows potential evidence of a shared genetic influence on GERD and CAD, suggesting it may contribute to the pathogenesis of both conditions through common biological mechanisms. This shared pathway highlights the possibility of genetic overlap and offers new perspectives on the interconnectedness of these complex diseases.

The findings of this study have significant clinical implications for both the management and prevention of cardiovascular disease in patients with GERD. Identifying GERD as an independent risk factor for CAD underscores the importance of monitoring GERD patients more closely for potential cardiovascular risks. Given the shared genetic mechanisms identified between GERD and CAD, this study suggests that interventions targeting GERD may not only improve gastrointestinal outcomes but could also have a beneficial effect on reducing the risk of cardiovascular events. For instance, early detection and management of GERD in at‐risk populations may offer an opportunity for proactive cardiovascular risk reduction, potentially through lifestyle modifications, pharmacologic treatments, or both. The shared genetic loci identified in our study, including those in IGF2BP1 and FAM19A5, offer promising candidates for further exploration as therapeutic targets that may simultaneously address both GERD and CAD, enhancing the precision of treatment strategies. In light of these findings, future research should prioritize exploring the biological pathways that link GERD and CAD, with particular emphasis on the inflammatory and metabolic pathways involved. Understanding the molecular mechanisms underlying the shared genetic loci between these conditions could pave the way for the development of targeted therapies. Additionally, clinical trials investigating the potential cardiovascular benefits of GERD management interventions could offer further insights into whether treating GERD directly influences CAD outcomes. Ultimately, these findings open new avenues for integrative approaches to disease management, suggesting that therapeutic strategies addressing both GERD and cardiovascular risk factors could improve patient outcomes across these interconnected conditions.

A key strength of this study is the use of MR, which leverages genetic variants as instrumental variables to infer causality, thereby minimizing confounding and reverse causation. Additionally, the use of multiple complementary approaches, including Cox regression model, MVMR, LDSC, MTAG, CPASSOC, ρ‐HESS and colocalization analysis, enhances the robustness of our findings. However, some limitations should be acknowledged. While GWAS summary data provides a powerful tool for genetic analysis, they may not capture all relevant genetic variation, particularly in non‐European populations. Furthermore, although we adjusted for major cardiovascular risk factors, other unmeasured confounders may still exist. Another limitation is the lack of gender‐specific analysis, as there was no gender‐specific GWAS datasets available for GERD or CAD at the time of this study. This absence restricted our ability to examine gender differences in the relationships between GERD and CAD, which may be a crucial factor in future investigations.

## 5 Conclusion

In conclusion, this study provides strong evidence that GERD is an independent causal risk factor for CAD and that shared genetic factors contribute to the association between these conditions. The identification of novel pleiotropic genes further enhances our understanding of the genetic basis of GERD and its relationship with cardiovascular disease. These findings underscore the importance of considering GERD in the context of cardiovascular risk assessment and highlight the potential for new therapeutic strategies targeting the shared genetic pathways between GERD and CAD.

## Author Contributions

Haozhang Huang and Jiaman Ou contributed to the conception or design of the work. Haozhang Huang performed the statistical analysis and revised the manuscript. Yiying Zhen and Jiaman Ou draft the manuscript. Haozhang Huang performed genetic analysis. Xiang Yuan validated the data and reviewed the paper. Min Ruan and Huan Lu revise the paper. Dakai Liang, Dehua Huang and Fengyang Deng supervise the study. All gave final approval and agreed to be accountable for all aspects of work ensuring integrity and accuracy.

## Ethics Statement

Publicly available GWAS summary data were utilized, eliminating the need for new ethical approvals.

## Conflicts of Interest

The authors declare no conflicts of interest.

## Supporting information


**Supplement Figure 1:**. Sensitivity analysis of the causal effect of GERD on CAD. **Supplement Figure 2:** rs4643373 between CAD and GERD. **Supplement Figure 3:** rs9615905 between CAD and GERD.

Supplement Table 1: Instrumental variables of GRED. Supplement Table 2: Instrumental variables of CAD. Supplement Table 3: Sensitivity analysis of bidirectional MR results of CAD and GRED Supplement. Table 4: Mediating effects of metabolic risk factors on the causal role of GERD in CAD. Supplement Table 5: Sensitivity analysis of MVMR results of coronary artery disease. Supplement Table 6: Significant pleiotropic SNPs identified by cross‐trait meta‐analyses. Supplement Table 7: Shared SNPs was analyze using LDlink between GERD and CAD. Supplement Table 8: Identification of local genetic correlations using ρ‐HESS.

## Data Availability

All GWAS data utilized in this study, including those generated or analyzed, are fully available within the published article and its accompanying Supporting Information.
