# Supplementary material for: Mapping Gastroesophageal Reflux Disease and Coronary Artery Disease: A Comprehensive Analysis of Multivariable Mendelian Randomization and Shared Genetic Etiology
Source: Clin Cardiol. 2025 Oct 21;48(10):e70213. doi: 10.1002/clc.70213 (PMC12538509; doi:10.1002/clc.70213)

**Supplement Figure 1. Sensitivity analysis of the causal effect of GERD on CAD.**


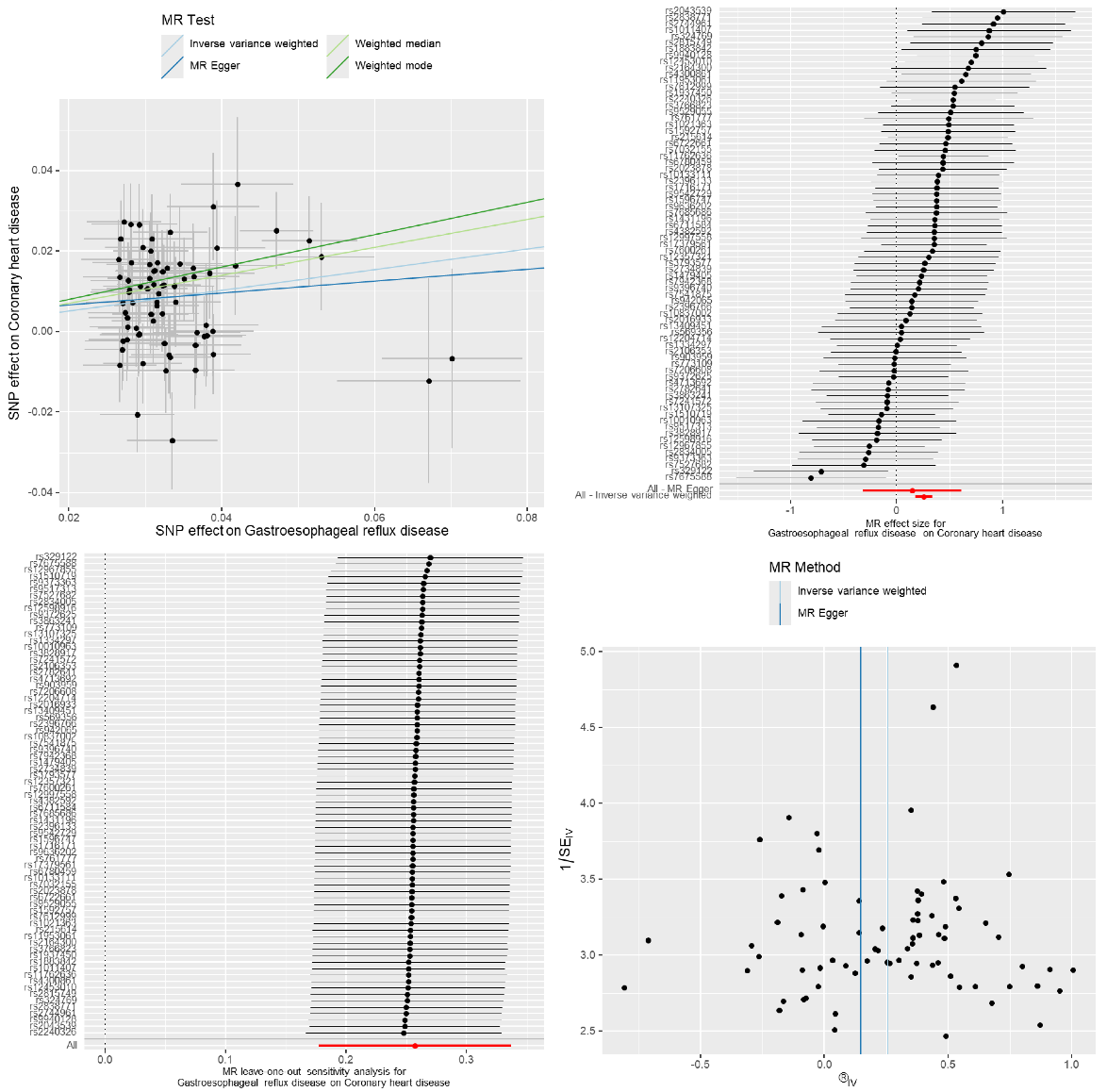


**Supplement Figure 2. rs4643373 between CAD and GERD.**


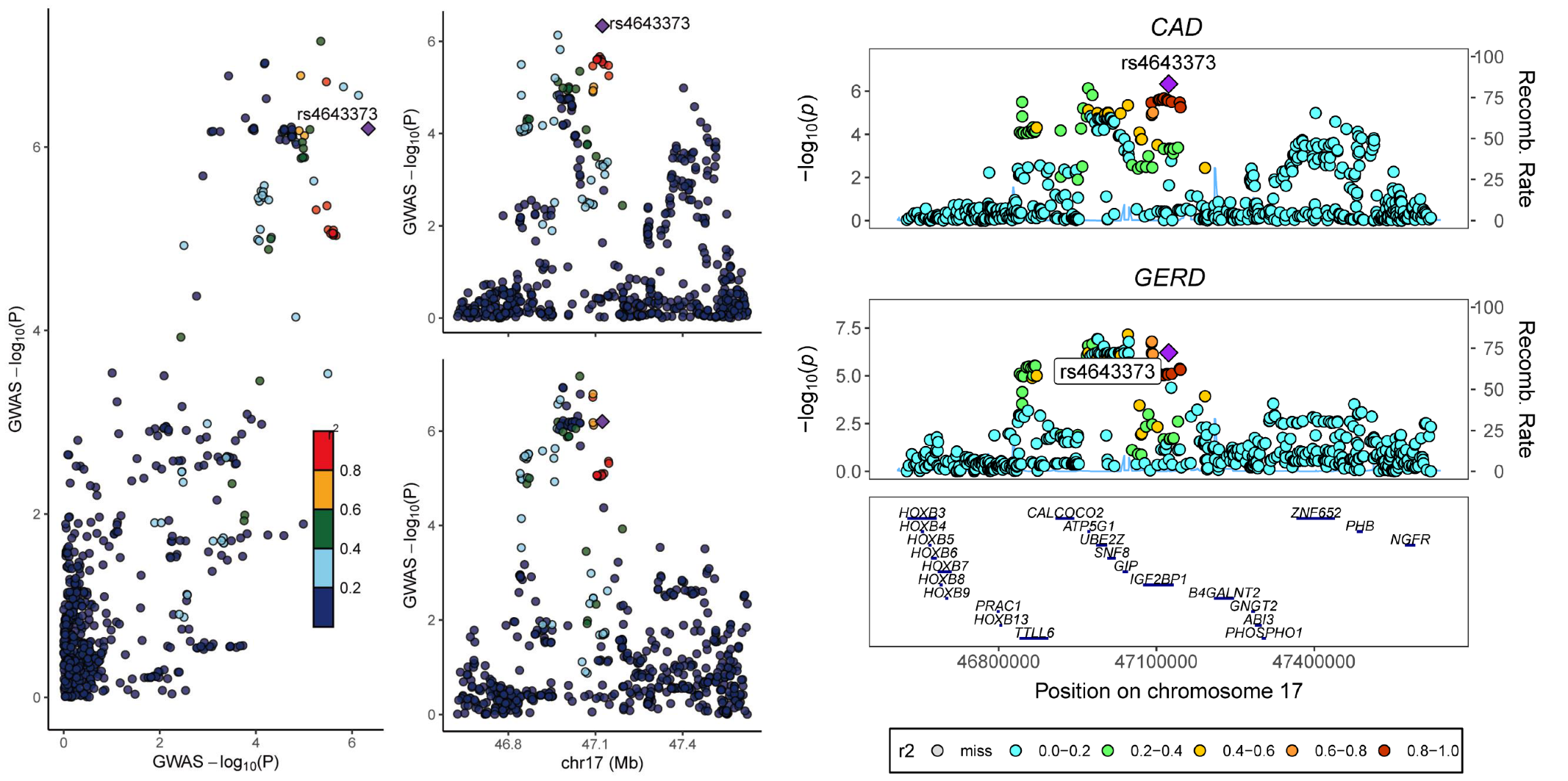


**Supplement Figure 3. rs9615905 between CAD and GERD.**


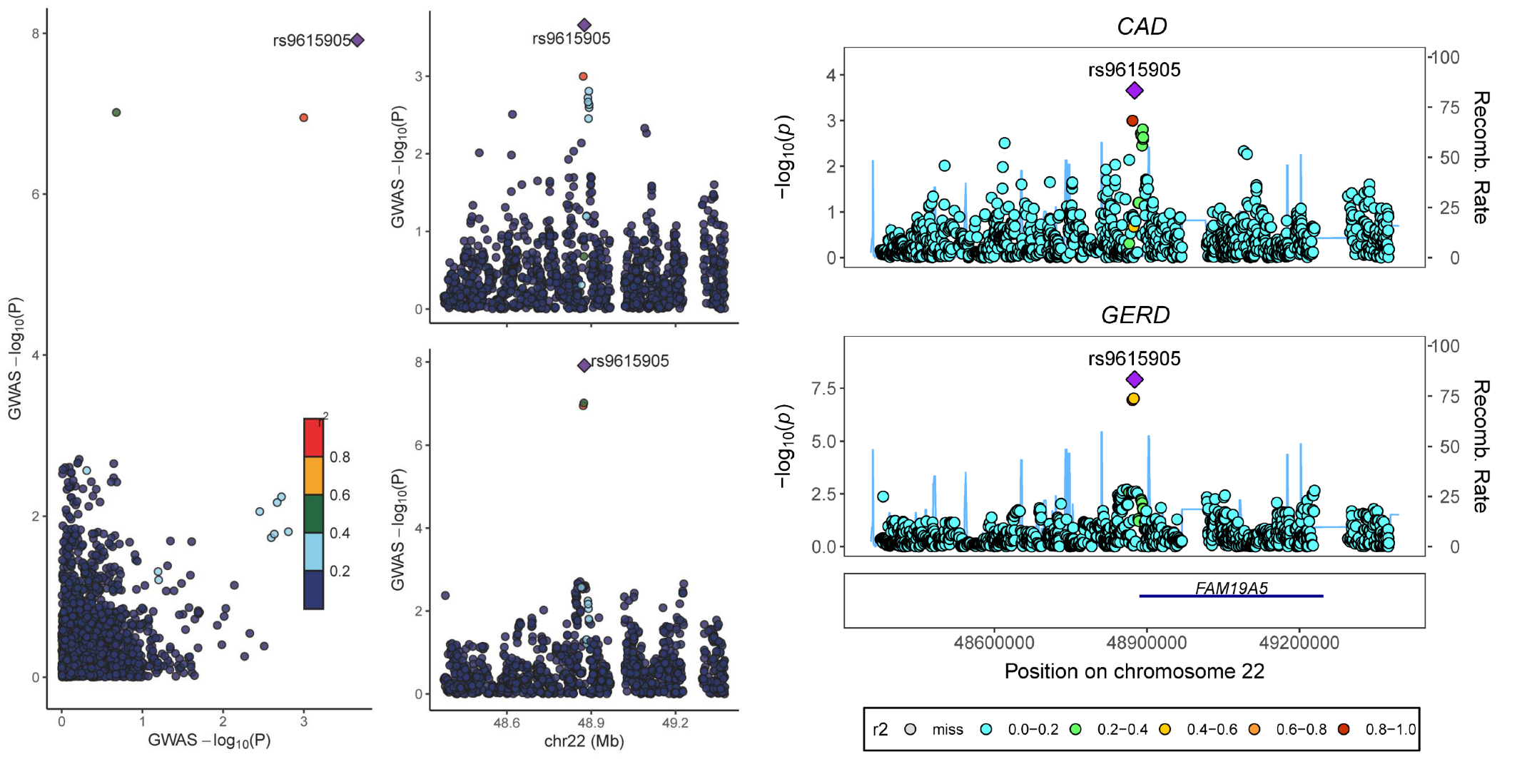

Supplement: Supplementary file 1 — Supplement Figure 1:. Sensitivity analysis of the causal effect of GERD on CAD. Supplement Figure 2: rs4643373 between CAD and GERD. Supplement Figure 3: rs9615905 between CAD and GERD. [file CLC-48-e70213-s002.docx]
